# Allergy and immunology in young children of Japan: The JECS cohort

**DOI:** 10.1016/j.waojou.2020.100479

**Published:** 2020-11-07

**Authors:** Kiwako Yamamoto-Hanada, Kyongsun Pak, Mayako Saito-Abe, Limin Yang, Miori Sato, Makoto Irahara, Hidetoshi Mezawa, Hatoko Sasaki, Minaho Nishizato, Kazue Ishitsuka, Yukihiro Ohya, Japan Environment and Children's Study (JECS) Group, Michihiro Kamijima, Shin Yamazaki, Yukihiro Ohya, Reiko Kishi, Nobuo Yaegashi, Koichi Hashimoto, Chisato Mori, Shuichi Ito, Zentaro Yamagata, Hidekuni Inadera, Takeo Nakayama, Hiroyasu Iso, Masayuki Shima, Youichi Kurozawa, Narufumi Suganuma, Koichi Kusuhara, Takahiko Katoh

**Affiliations:** a Allergy Center, National Center for Child Health and Development, Tokyo, Japan; b Medical Support Center for the Japan Environment and Children's Study, National Research Institute for Child Health and Development, Tokyo, Japan; c Division of Biostatistics, Department of Data Management, Center for Clinical Research and Development, National Center for Child Health and Development, Tokyo, Japan

**Keywords:** Asthma, Atopic dermatitis, Atopic march, Children, Eczema, Epidemiology, Food allergy, Kawasaki disease, Primary immune deficiency, Wheeze, Atopic dermatitis, AD, ISAAC, The International Study of Asthma and Allergies in Childhood, Kawasaki disease, KD, food protein-induced enterocolitis syndrome, FPIES, University Hospital Medical Information Network, UMIN, primary immunodeficiency disorder, PID, GI, gastrointestinal

## Abstract

**Background:**

Capturing epidemiological signatures is essential to document burdens of disease and to design health care services, including prevention measures, clinical interventions, and policies. There are large geographical and ethnic variations in the epidemiology of allergic and immunological diseases. Various data are available from North America and Europe, but the epidemiology of allergic and immunological diseases in Asia is not well documented.

**Objective:**

To characterize epidemiological signatures of allergic and immunological disease in young children in Japan.

**Methods:**

This was a national, multicenter, prospective birth cohort study: Japan Environment and Children's Study (JECS). A general population of 103,060 women was enrolled during pregnancy. Allergic and immunological outcomes were assessed among young children using questionnaire data.

**Results:**

The prevalence of caregiver-reported immediate food allergy was 7.6%, 6.7%, and 4.9% at age 1, 2, and 3 years, respectively. Hen egg allergy was most common (5.4% prevalence at age 1 year) followed by allergies to cow milk and wheat. Several patterns of allergic symptom clusters were identified. Physician diagnosed, as reported by the caregiver, non-IgE mediated gastrointestinal food allergy affected 0.5% of infants. By contrast, caregiver-reported gastrointestinal food allergies affected 1.4% of children. Kawasaki disease affected 0.3% and 0.4% children, respectively, at age 1 and 3 years. Primary immunodeficiency disorders affected 0.005% children at age 3 years.

**Conclusion:**

These data provide important epidemiological signatures of allergy and immunology in young Japanese children including the age-specific prevalence of allergic disease, Kawasaki disease, and primary immune deficiency.

## Introduction

Capturing epidemiological signatures is essential to document burdens of disease and to design health care services, including prevention measures, clinical interventions, and policies. Epidemiological changes can affect the economic burden of disease and economic growth. Multiple epidemiological studies have examined allergic features and found a highly variable prevalence of allergic diseases across countries and ages.^1^ Allergic diseases such as asthma are likely to result from complex interactions between genetic factors and environmental exposures.^2^^,^^3^

Early events during the lives of young children influence later life course trajectories.^4^ Prescott et al noted that individuals of Asian ethnicity were more susceptible to allergic diseases.^5^ A food allergy pandemic has been observed globally,^6^ and children of Asian/Indian/Pacific Islander origin were more likely than other children to be admitted to intensive care units for fatal food-triggered anaphylaxis in North America.^7^ In Tokyo, Japan, more than 70% of children from an inner-city local birth cohort showed IgE sensitization at age 9 years old, as measured by Immuno Solid-phase Allergen Chip, suggesting that the majority of Japanese children were atopic.^8^ Furthermore, about 32% of school aged children were currently experiencing rhinitis in a recent study conducted in Tokyo.^8^

Neonatal and infantile gastrointestinal (GI) allergies arising from non-IgE-mediated (cell-mediated) mechanisms have dramatically increased in prevalence in recent decades in Japan.^9^ Baker et al^10^ calculated that 0.5% children may be affected by food protein-induced enterocolitis syndrome (FPIES) globally. Moreover, the incidence rates of Kawasaki disease (KD) in Asian countries (especially Japan, Korea, and Taiwan) are higher than those in North America and Europe.^11^ To mitigate the pandemic of allergic and immunological diseases, physicians and scientists must work together, and the starting point for such efforts is epidemiological data describing these diseases.

Therefore, understanding global allergic and immunological signatures in young children at an epidemiological level is vitally important.

Various epidemiological data are available from North America and Europe, but the epidemiology of allergic and immunological diseases in Asia is not well documented.

No epidemiological studies of general populations across Japan have examined allergic and immunological features. We sought to characterize epidemiological signatures of allergic and immunological disease in young children in Japan using a general population national birth cohort.

## Methods

### Study design, setting, and population

This was a nationwide, multicenter, prospective birth cohort study: the Japan Environment and Children's Study (JECS).^12^ JECS is an ongoing cohort study conducted by the Ministry of the Environment, Japan.^13^^,^^14^ A general population of 103,060 pregnant women was enrolled in the JECS in 15 study areas, covering a wide geographical area from the north of the country (Hokkaido) to the south (Okinawa), from January 2011 to March 2014. All participants within each of the 15 study areas were followed. Eligibility criteria were as follows: 1) currently pregnant; 2) living in the study area for the foreseeable future; 3) expected delivery between August 1, 2011 and mid-2014; and 4) ability to understand the Japanese language. In total, 104,062 newborns were enrolled in the JECS. The registry of the JECS is the University Hospital Medical Information Network (UMIN). JECS protocols for the main study and the sub-cohort study are described on the website of the Japanese Ministry of the Environment.^15^^,^^16^

### Ethics statement

The JECS protocol was reviewed and approved by the Ministry of the Environment Institutional Review Board on Epidemiological Studies and by the ethics committees of all participating institutions. Written informed consent was obtained from all participants. The JECS was conducted in accordance with the principles laid out in the Helsinki Declaration and other national regulations and guidelines.

### Data source: questionnaire

Written questionnaires were provided to caregivers during pregnancy and when the offspring were age 6 months,1, 1.5, 2, 2.5, and 3 years. Caregivers answered questions regarding their child and their family. Data input was conducted at 15 study areas. Data management was performed by the study program office.

### Variables: outcomes

Information on each child's background, lifestyle, and nutrition was assessed using questionnaires in Japanese. Caretaker reports of physician diagnoses of diseases [atopic dermatitis (AD), asthma, food allergy, allergic rhinitis and conjunctivitis, GI allergy (non-IgE mediated food allergy), primary immunodeficiency disorder (PID) and KD] were obtained from questionnaires. Definitions of caretaker-reported, physician-diagnosed outcomes are shown in Table S3. Caregiver-reported wheeze, eczema/AD and rhinitis symptoms were evaluated using the questionnaire of the International Study of Asthma and Allergies in Childhood (ISAAC).^17^ Caregiver-reported immediate food allergy and GI allergy (non-IgE-mediated food allergy) were defined as a history of reactions to causal foods with avoidance (Table S4). Both were evaluated using questionnaires.

### Bias and study size

Data were collected prospectively from a nationwide birth cohort, minimizing many forms of bias. No sample size estimate was calculated given the very large number of participants (>100,000) in the study.

### Statistical analyses

Questionnaire data from 92,945 singleton mothers and 48,081 fathers who were enrolled in the study and did not withdraw consent were used for analysis (Fig. S1). We also analyzed a fixed data set (jecs-ta-201,901,930-qsn, released October 2019). For all analysis populations, summary statistics were prepared as follows. For continuous variables, the minimum, 25th, 50th, and 75th percentiles and maximum values were calculated. For categorical variables, frequencies and percentages were calculated. In addition, frequencies and percentages of missing values were calculated for all variables except for checkbox variables. The frequencies and percentages of children experiencing each disease/symptom were shown in Venn diagrams and Upset plots to visualize allergic march and the intersections of multiple conditions (Fig. 3, Fig. 4, S2 and S3). All analyses were descriptive and no adjustment for confounders was performed. Missing values were not imputed. Because parents answered questions only if their children had certain outcomes, the number of missing values for each outcome was uncertain.

Sensitivity analysis was not performed.R statistical software version 3.6.2 for Windows was used for all statistical analyses.

## Results

### Baseline characteristics

Table S1 shows the baseline characteristics of the cohort. Maternal mean age at recruitment was 30.7 years. Allergic medical history was common among mothers (hay fever, 36.1%; atopic dermatitis, 15.8%; and asthma, 11.0%). Maternal educational background varied. Both low-income and high-income families were included based on annual household income data. In terms of solid food introduction (Table S2), around 5% of infants started consuming hen eggs before 6 months of age. Only 0.1% of infants had started consuming peanuts by 6 months of age. Fish was most often introduced into the diet around 7–8 months of age (48.6%). The median duration of breastfeeding was 14 months.

### Caregiver-reported physician diagnosis of allergic and immunological diseases

**Fig. 1:**
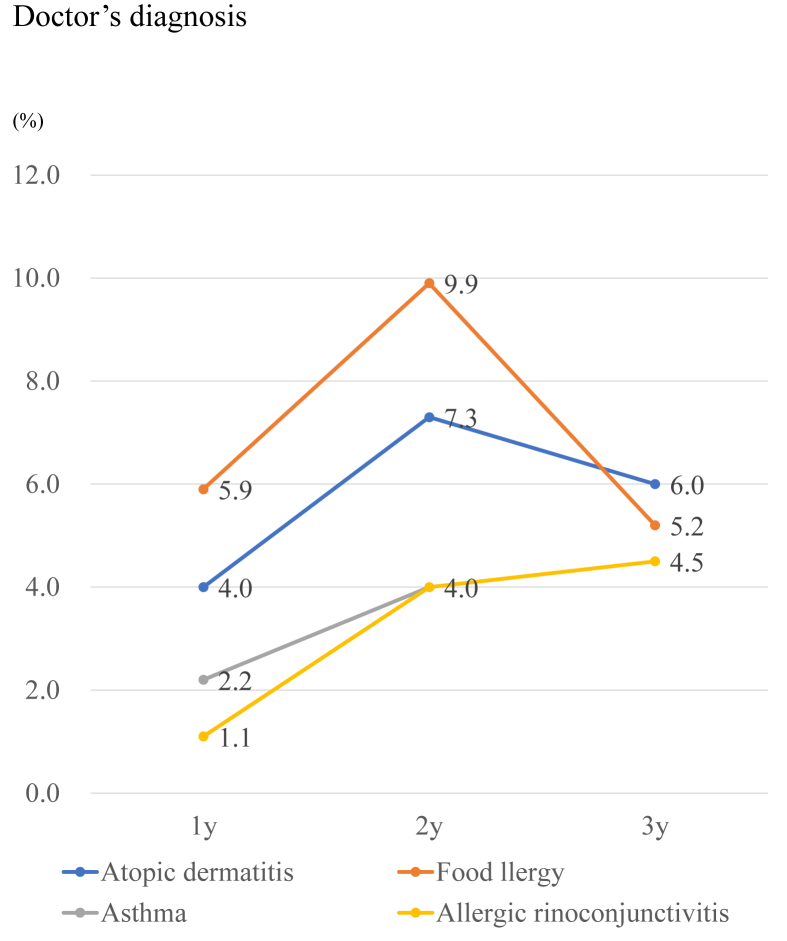
Prevalence of caregiver-reported physician diagnosis of allergic disease

**Fig. 2:**
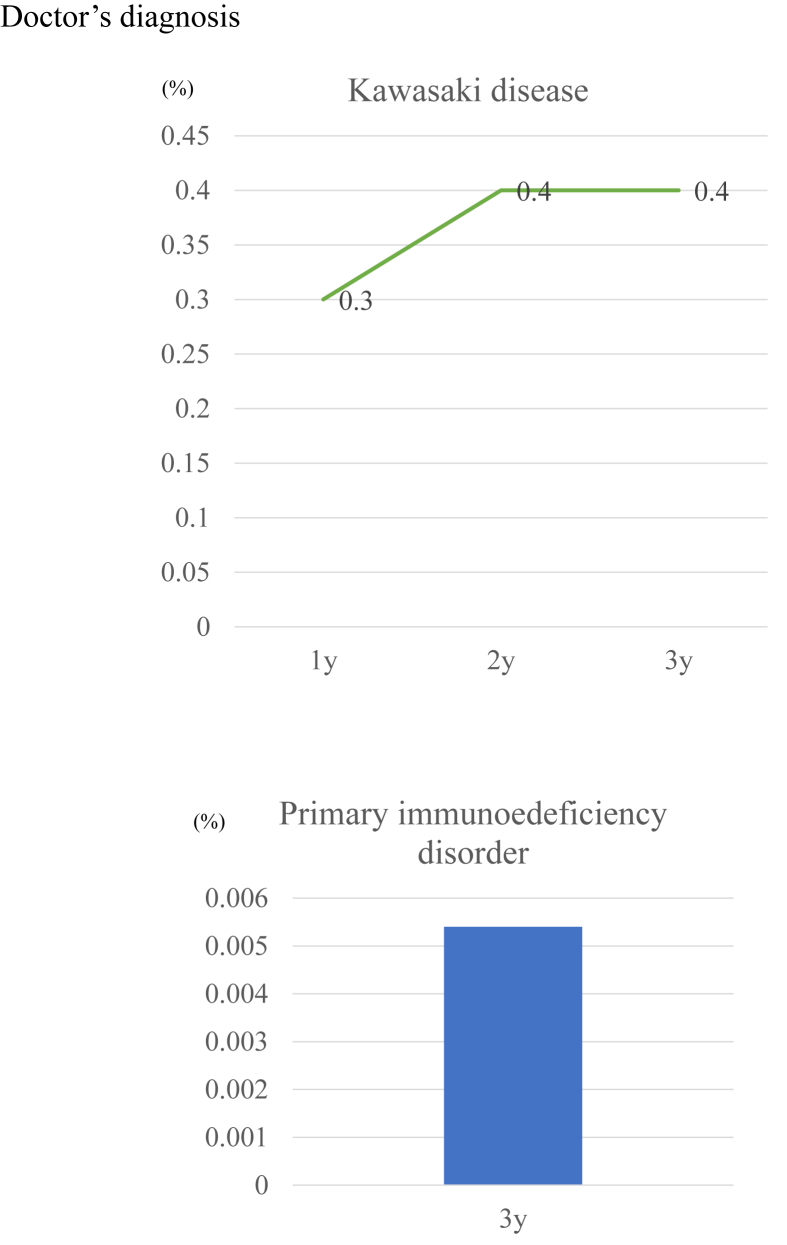
Prevalence of Kawasaki disease and primary immunodeficiency

The prevalence of caregiver reports of a physician diagnosis of food allergy was 5.9%, 9.9%, and 5.2% at age 1, 2, and 3 years, respectively (Fig. 1). The prevalence of caregiver-reported, physician-diagnosed food allergy and atopic dermatitis peaked at 2 years of age. By contrast, the prevalence of caregiver-reported, physician-diagnosed asthma and allergic rhinitis increased with age (both 4.5% at age 3 years). The prevalence of caregiver-reported, physician-diagnosed KD was 0.3% and 0.4%, respectively, at age 1 and 3 years (Fig. 2). PIDs affected 0.005% of children by age 3 years.

### ISAAC-based allergic features (eczema, wheeze, and rhinitis) self-reported by caregivers

The prevalence of eczema decreased with age (16.8% at age 1 year and 13.4% at age 3 years) (Table 1). One or more nights per week of sleep disturbance resulting from eczema occurred in 0.9%–2.0% of children prior to 3 years of age. The peak prevalence of wheeze was 20.6% at 2 years of age. About 2% of children suffered from wheeze-associated sleep disturbances one or more nights per week at age 3 years. The prevalence of rhinitis was 24.8% and 25.3% at age 2 and 3 years, respectively (nasal symptoms were not evaluated at age 1 year). Rhinitis interfered with the daily activities of about 5% of children at age 2 and 3 years.

**Table 1:** Allergic features (eczema, wheeze and rhinitis symptoms) self-reported by caregivers

**Skin (N = 92,945)**		**1 year, n (%)**	**2 years, n (%)**	**3 years, n (%)**

Current eczema	Yes	15,639 (16.8)	14,245 (15.3)	12,411 (13.4)
Onset of eczema	Before 2 months	3655 (3.9)	–	–
	2–5 months	7476 (8.0)	–	–
	6–8 months	2927 (3.1)	–	–
	9–12 months	1697 (1.8)	–	–
Complete eczema remission	Yes	10,457 (11.3)	9305 (10.0)	7822 (8.4)
	No	5329 (5.7)	5262 (5.7)	4853 (5.2)
Sleep disturbance caused by eczema	Never in the past 12 months	10,921 (11.7)	10,621 (11.4)	8813 (9.5)
	Less than one night per week	3553 (3.8)	3302 (3.6)	2973 (3.2)
	One or more nights per week	1837 (2.0)	748 (0.8)	876 (0.9)
Dry skin	Yes	32,126 (34.6)	32,798 (50.3)	32,746 (46.8)
**Respiratory (N** = **92,945)**		**1 year, n (%)**	**2 years, n (%)**	**3 years, n (%)**
Current wheeze	Yes	16,194 (17.4)	19,101 (20.6)	13,288 (14.3)
Frequency of wheeze	None	5383 (5.8)	7497 (8.1)	5239 (5.6)
	1–3 times	9583 (10.3)	12,152 (13.1)	9737 (10.5)
	4–12 times	2608 (2.8)	2904 (3.1)	2067 (2.2)
	13 times or more	928 (1.0)	472 (0.5)	379 (0.4)
Sleep disturbance caused by wheeze	Never woken by wheezing	9165 (9.9)	12,257 (13.2)	9736 (10.5)
	Less than one night per week	5507 (5.9)	7355 (7.9)	5206 (5.6)
	One or more nights per week	2665 (2.9)	2270 (2.4)	1567 (1.7)
Speech difficulty caused by wheeze	Yes	991 (1.1)	871 (0.9)	866 (0.9)
Wheeze caused by physical activity and crying	Yes	2459 (2.6)	1775 (1.9)	1767 (1.9)
Nighttime cough	Yes	11,183 (12.0)	6458 (6.9)	6232 (6.7)
**Nose and eyes (N** = **92,945)**		**1 year, n (%)**	**2 years, n (%)**	**3 years, n (%)**
Current rhinitis	Yes	–	23,077 (24.8)	23,496 (25.3)
Eye symptom	Yes	–	2408 (2.6)	3060 (3.3)
Rhinitis interferes with child's daily activities	Not at all	–	5162 (5.6)	5078 (5.5)
	A little	–	12,942 (13.9)	14,174 (15.2)
	A moderate amount	–	4343 (4.7)	3972 (4.3)
	A lot	–	928 (1.0)	739 (0.8)

-, not evaluated

### Immediate food allergies reported by caregivers

The prevalence of caregiver-reported immediate food allergy was 7.6%, 6.7%, and 4.9% at age 1, 2, and 3 years, respectively (Table 2). Hen egg allergy was the most common (5.3% at age 1 year) followed by allergy to cow milk (2.1% at age 1 year) and wheat (0.5% at age 1 year). The prevalence of allergy to hen egg, cow milk and wheat decreased with age. The prevalence of allergy to fish, soy, fruit, crustaceans, buckwheat, sesame, nuts, and peanuts is shown in Table 2.

**Table 2:** Prevalence of caregiver-reported immediate food allergy (N = 92,945)

	1 year, n (%)	2 years, n (%)	3 years, n (%)
Caregiver report of physician-diagnosed food allergy	5515 (5.9)	9224 (9.9)	4873 (5.2)
Caregiver-reported immediate food allergy^a^	7018 (7.6)	6236 (6.7)	4511 (4.9)
Hen egg	4924 (5.3)	4350 (4.7)	2966 (3.2)
Cow milk	1921 (2.1)	1543 (1.7)	953 (1.0)
Wheat	491 (0.5)	342 (0.4)	208 (0.2)
Soy	205 (0.2)	130 (0.1)	73 (0.1)
Fish	292 (0.3)	288 (0.3)	259 (0.3)
Fruit	351 (0.4)	329 (0.4)	308 (0.3)
Crustacean	309 (0.3)	396 (0.4)	337 (0.4)
Buckwheat	98 (0.1)	159 (0.2)	167 (0.2)
Sesame	67 (0.1)	79 (0.1)	78 (0.1)
Nut and peanut	165 (0.2)	300 (0.3)	446 (0.5)

a After eating certain foods, children may have symptoms such as repeated vomiting, bloody stools, diarrhea, and weight loss occurring 3 h to several days after consumption. Immediate food allergy excludes food poisoning, gastroenteritis resulting from infection, and overeating

### GI food allergies (non-IgE mediated food allergies

GI food allergies affected 0.5% of children (Table 3). Caregiver-reported GI food allergy symptoms, however, occurred in 1.4% children. Among children with GI allergy symptoms (n = 1437), the median age at symptom onset was 9 months. Frequent vomiting affected 46.9% of children (n = 632). The most common trigger food was hen egg (34.5%, n = 465) followed by cow milk (21.7%, n = 333).

**Table 3:** Characteristics of gastrointestinal allergy (non-IgE mediated food allergy) at age 1.5 years (N = 92,945)

Gastrointestinal allergy, n (%)	Yes	476 (0.5)
Caregiver's report		
Any symptoms^a^, n (%)	Yes	1347 (1.4)
Symptoms, n	Frequent vomiting	632
	Bloody stool	106
	Frequent diarrhea	662
	Failure to thrive	89
	Other	214
Onset of gastrointestinal allergy, age in months	Minimum	0
	25% tile	6
	50% tile	9
	75% tile	12
	Maximum	29
Causal allergen, n	Cow milk	333
	Hypoallergenic milk	18
	Breast milk	89
	Rice	18
	Soy	76
	Wheat	75
	Hen egg	465
	Other	424
	Unknown	99

a After eating certain foods, children may have symptoms such as repeated vomiting, bloody stools, diarrhea, and weight loss occurring 3 h to several days after consumption. Immediate food allergy excludes food poisoning, gastroenteritis resulting from infection, and overeating

### Atopic march and clustering of allergic symptoms

**Fig. 3:**
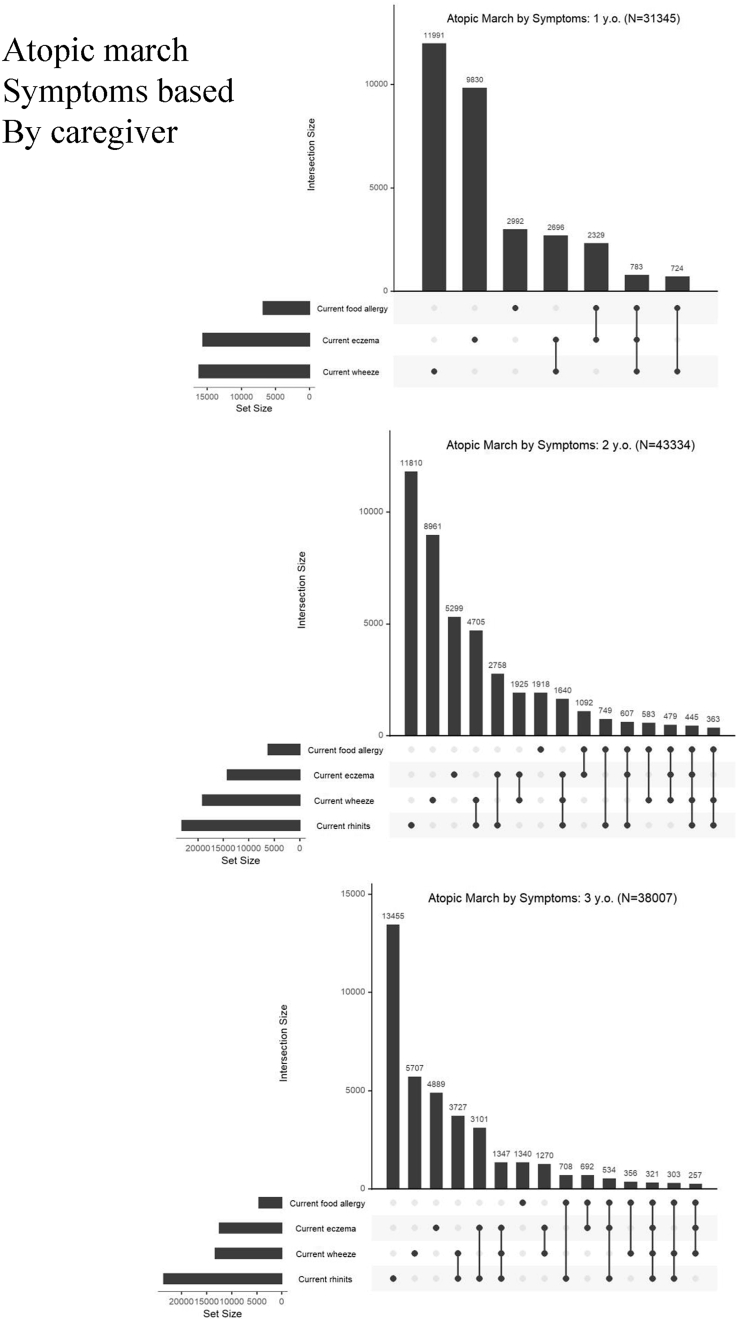
Allergic symptom combinations recorded on caregiver questionnaires

**Fig. 4:**
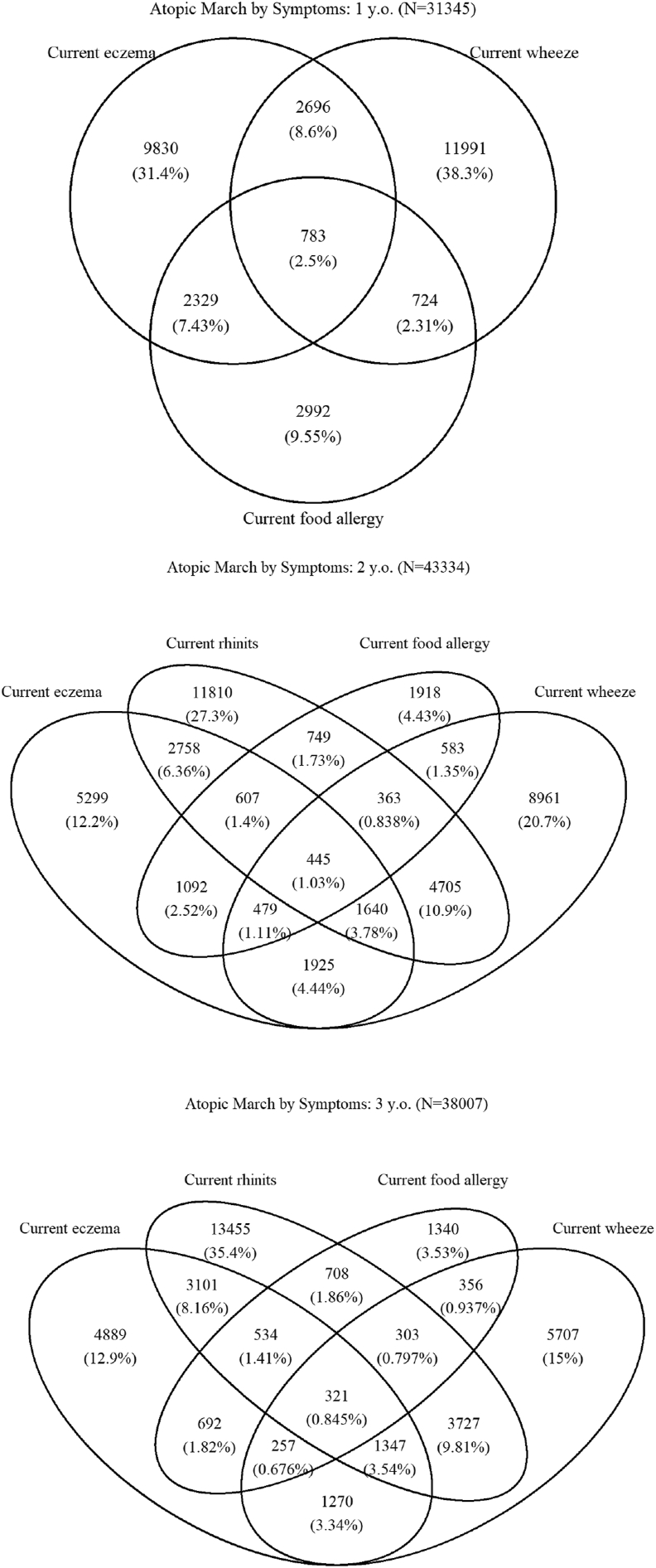
Venn diagram of allergic symptom combinations (eczema, food allergy, wheeze, and rhinitis) recorded on caregiver questionnaires

As expected for atopic march (Fig. 3, Fig. 4), clustering of eczema, wheeze, food allergy, and rhinitis in young children was common as recorded by caregivers. The most common combination of allergic symptoms was eczema and wheeze (8.6%) followed by wheeze and eczema (7.43%) at 1 year of age. In addition, 2.5% of 1-year-old infants had 3 allergic symptoms: wheeze, eczema, and food allergy. At age 3 years, the most common combination of allergic symptoms was wheeze and rhinitis (9.81%), followed by rhinitis and eczema (8.61%) and wheeze and rhinitis (3.54%). The prevalence of allergic symptom clusters confirmed by physician diagnosis was lower than caregiver-reported prevalence (Figs. S2 and S3).

## Discussion

In this study, we described the epidemiological signatures of allergic and immunological diseases (allergic diseases, KD, and PIDs) in young Japanese children using data from a national birth cohort study.

All epidemiological studies, including this one, have limitations. Reporting biases inevitably arose in our study. First, outcome assessments were not made directly by clinicians, but through a questionnaire given to caregivers. There is no linkage system for medical record review in Japan. Parents answered only if their children had certain outcomes, so missing values for each outcome are uncertain. Inability to determine missing data/response rates for some outcomes means that bias could have influenced the study findings. Thus, the prevalence and incidence of disease might have been underestimated. Second, information regarding medical interventions, including medications, was not obtained. Third, detailed diagnoses of congenital immunodeficiencies were unclear and some may have been misclassified. Finally, food allergy was not defined using oral food challenge tests.

A strength of the study we captured general populations from a variety of areas from across Japan in the nationwide birth cohort, minimizing selection bias. Previously, Michikawa et al^18^ showed that our study population's characteristics were similar to those of the Japanese population using vital statistics from the Japanese government. The results of the JECS provide national representative epidemiological signatures of allergic and immunological disease. We followed participants over time with high rates of participation and low losses to follow-up. Furthermore, we enrolled a large cohort of more than 100,000 participants and this sample size provided high statistical power.

### Eczema (AD)

Eczema is considered the beginning of the atopic march.^19^ A systematic review of epidemiological studies conducted between 1990 and 2000 found that the prevalence of eczema had increased in eastern Asia, Africa and some European countries.^20^ Most studies assessed AD prevalence only among children 6 years and older, and the global prevalence of eczema in younger children remains unclear. However, eczema prevalence in young infants tends to be higher compared with older children. The incidence of eczema over the first year of life was reported as 27.9% among children in Tokyo, Japan. The prevalence of AD at 18 months of age was 15% in a Danish cohort.^21^ A UK study also demonstrated that the prevalence of AD in 3-month-old infants was 24.3%.^22^ In our study of children across Japan, the incidence of eczema over the first year of life (16.8%) was lower compared with data from Tokyo and the United Kingdom. The prevalence of eczema was higher in African countries and in urbanizing areas.^20^ Several cohort studies have indicated that AD has several phenotypes and that a majority of AD cases develop before age 5 years.^23^, ^24^, ^25^ We expect that JECS will characterize several phenotypes of eczema using longitudinal data and investigate risk factors for AD.

Sleep disturbances resulting from eczema symptoms occurred in 0.9%–2.0% of young children in Japan. In a UK interventional study (BEEP Study),^26^ 1%–2% children suffered from severe eczema at age 2 years. Growth rates are lower in children with eczema.^27^

### Wheeze and asthma

Wheeze is the most common symptom of asthma in young children.^30^ Various therapies for uncontrolled asthma have been developed over the past few decades. According to another survey,0.23% of all adolescents had severe asthma in a Swedish population.^31^ In an Italian population, severe asthma affected 0.1% of individuals 6 years and older.^32^ In Japan, the number of children hospitalized because of asthma exacerbation decreased from 1996 to 2014.^33^ It is possible that asthma control has been improving over time. However, about 1/100 young children in our study suffered from sleep disturbances because of wheeze 1 or more nights per week until the age of 3 years. Our study highlights unmet needs in wheeze and asthma management for young children in Japan.

### Rhinitis

Rhinitis is quite common globally. The Phase III ISAAC study reported that the prevalence of allergic rhinitis was 0.8%–14.9% among 6- to 7-year-old children. In our study, the prevalence of rhinitis was 24.8% and 25.3% at 2 and 3 years of age, respectively. Rhinitis interfered with the daily activities of about 5% of children at age 2 and 3 years. In a birth cohort in Tokyo about a decade ago, rhinitis affected 10.6% of children aged 5 years.^8^ In Taiwan, about 50% of school children suffered from allergic rhinitis.^34^ We previously reported that the prevalence of allergic rhinitis in 9-year-old children was 31.2%, and Cri *j 1* (Japanese cedar) and Der *f 1* (mites) sensitization were observed in 57.8% and 54.3% of individuals, respectively, from the JECS.^8^ It appears that the prevalence of rhinitis has increased in Japan.

### Immediate food allergy

The prevalence of caregiver-reported, physician-diagnosed food allergy was 5.9%, 9.9%, and 5.2% at age 1, 2, and 3 years, respectively. By contrast, the prevalence of caregiver self-reported immediate food allergy was 7.6%, 6.7%, and 4.9% at age 1, 2, and 3 years, respectively. The 3 most common food allergens were hen egg, cow milk, and wheat. Peanut and nut allergies were uncommon among Japanese children. Trends in terms of allergens were similar to those described by past reports in Japan.^35^^,^^36^ We speculate that caregiver-reported, physician-diagnosed food allergies may include immediate food allergies as well as GI allergies such as FPIES. The peak age for caregiver reports of physician-diagnosed food allergy was 2 years. As introduction of allergenic foods in infants tends to be delayed, the timing of diagnosis might also be delayed even though IgE sensitization has already developed. Shoda et al^36^ reported that the prevalence of food allergy decreased from 9.0% at age 1 year to 7.5% at age 3 years in a birth cohort in Tokyo (T-Child Study). These data suggested that certain children could achieve remission from food allergy before 3 years of age. The EuroPrevall birth cohort study conducted in European countries showed that the prevalence of hen egg allergy was 0.07%–2.18% in young infants using food challenge tests.^37^ The prevalence of hen egg allergy differed among countries. According to a systematic review of the prevalence of food allergy in European countries, the prevalence of self-reported food allergy was 1.6%–38.7% among 2- to 5-year-old children. Although the prevalence of food allergy differed among studies, we believe that the prevalence of food allergies in Japan is higher than in other countries.^38^ In the United States, Asian populations tend to have higher prevalence of food allergies compared with individuals of other ethnicities.^39^ There may be a genetic component to food allergy risk.

A systematic review by Moonesinghe et al^40^ reported that the prevalence of fish allergy ranged from 0% to 7%. Our study showed that the prevalence of fish allergy was 0.3%–0.5%. Because introduction of fish into the infant diet is traditional in Japan, the prevalence of fish allergy might be lower compared with other countries. Unfortunately, we were unable to distinguish between nut and peanut allergies because the general Japanese public tends to equate these two foodstuffs.

### Atopic march

At age 1 year, the most common combination of allergic symptoms was eczema symptoms and wheeze, followed by wheeze and eczema symptoms. At age 3 years, the most common combination of allergy symptoms was wheeze and rhinitis symptoms, followed by rhinitis and eczema symptoms and then by eczema symptoms, wheeze and rhinitis symptoms. Paller et al^19^ found that atopic combinations were variable, suggesting that young children could have several phenotypes of allergic features. We identified various patterns of allergic symptom clusters in young children, suggesting that atopic march is heterogeneous. UK birth cohorts evaluating children aged 1–11 years identified 8 phenotypes of allergic disease.^41^ As endotypes and phenotypes differed by ethnicity and environments,^42^, ^43^, ^44^,^42^, ^43^, ^44^ future longitudinal data from the JECS will identify such phenotypes in Japanese children. Although we identified allergic symptom clusters based on caregiver-reported physician diagnosis, the prevalence of these phenotypes differed when outcome assessment was conducted by caregivers. Most children do not typically see a doctor for rhinitis and wheezing because these symptoms can be treated with over the counter medications.

### Non-IgE mediated gastrointestinal food allergies

Caregiver-reported, physician-diagnosed gastrointestinal (GI) food allergies affected 0.5% of infants in Japan. By contrast, caregiver self-reported GI food allergies occurred in 1.4% of children. In our questionnaire survey, it was difficult to categorize phenotypes of FPIES. We did not ask questions related to the specific names of GI allergies such as FPIES, food protein-induced allergic proctocolitis, or food protein-induced enteropathy.^45^ Our data suggest that certain cases may not be diagnosed correctly. Recently, epidemiological data on FPIES have become available globally. Nowak-Wegrzyn et al^46^ reported an estimated prevalence of FPIES in US children of 0.51% using a cross-sectional, population-based survey; their results suggested that Asian children were more likely to be affected by FPIES. In a Spanish prospective study, the estimated cumulative incidence of FPIES was 0.7% among children.^47^ In Australia, the incidence of acute FPIES in infants under 2 years of age was 15.4/100,000/year in a population-based study.^48^ Although Blackman et al^49^ reported that common trigger foods were grains (88%) and cow milk (49%) in children with FPIES in a retrospective study conducted in the United States, our data showed that common trigger foods were hen egg and cow milk. Shimomura et al^50^ demonstrated 3 cases of FPIES caused by egg yolk in Japanese children. Our study also suggested that GI food allergies in Japan may have different phenotypes compared with Western countries.^51^ Increased awareness of FPIES should be a priority for the public.

### Primary immune deficiency

Primary Immune Deficiency (PID) is rare: the prevalence of clinically diagnosed PID is estimated 81.6 cases per 100,000 people in the US.^52^ The prevalence of clinically diagnosed PID within the first 3 years of life was five cases per 92,945 live births in the XXX. Ichimura et al.^53^ reported a PID prevalence of 2.3 cases per 100,000 people in a Japanese database study. Although our results were similar to those of a Japanese Patient Registry survey, the prevalence of PID in Japan appears to be lower than that in the US. Bousfiha et al.^54^ reviewed the worldwide prevalence and incidence of PID. They concluded that PID was more common than previously thought, occurring in up to 1 in 1200 people worldwide. This potential gap between the true situation and the assumptions of the medical community are concerning. Our results suggest that we need to improve the diagnosis and management of PID.

### Kawasaki disease

Japan has the highest prevalence of Kawasaki Disease (KD) in the world.^11^ KD is an acute systemic inflammatory illness that sometimes leads to coronary artery aneurysms, myocardial infarction, and sudden death in previously healthy young children.^55^ In North America, KD occurs primarily in individuals of Asian and Pacific Islanders heritage.^56^ In Japan, the annual incidence of KD is increasing and the disease affects approximately 300/100,000 children under 4 years of age according to a nationwide patient registry survey.^57^ The incidence of KD in the JECS showed the same tendency as the nationwide registry survey. Although the incidence of KD is also increasing in Japan, the etiology of KD has remained unclear since Dr. Tomisaku Kawasaki first identified KD in 1967.^55^ No prevention strategy has been discovered. We hope to clarify the mechanisms underlying KD in future studies.

## Conclusions

This study highlighted the allergic and immunological epidemiological signatures of allergic disease, KD, and PID in young children in Japan using data from a national birth cohort study. These data identified several clusters/groups of symptoms of allergic features that differ from the patterns observed in Western countries.

## Funding

This study was funded and supported by the 10.13039/501100006120Ministry of the Environment, Japan.

## Availability of data and materials

The data and materials used to derive our conclusions are unsuitable for public deposition due to ethical restrictions and specific legal framework in Japan. It is prohibited by the Act on the Protection of Personal Information (Act No. 57 of May 30, 2003, amended on September 9, 2015) to publicly deposit data containing personal information. The Ethical Guidelines for Epidemiological Research enforced by the Japan Ministry of Education, Culture, Sports, Science and Technology and the Ministry of Health, Labour and Welfare also restrict the open sharing of the epidemiologic data. All inquiries about access to data should be sent to jecs-en@nies.go.jp. The person responsible for handling inquiries sent to this e-mail address is Dr Shoji F. Nakayama, JECS Programme Office, National Institute for Environmental Studies.

## Author contributions

KY and YO contributed to the study design. KY, PK and YO contributed to the statistical plan. PK analyzed the data and prepared study results. All co-authors contributed to the interpretation of findings. KY led the drafting of the manuscript. All co-authors contributed to revising the manuscript and approved the final version.

Members of the JECS Group as of 2020: Michihiro Kamijima (principal investigator, Nagoya City University, Nagoya, Japan), Shin Yamazaki (National Institute for Environmental Studies, Tsukuba, Japan), Yukihiro Ohya (National Center for Child Health and Development, Tokyo, Japan), Reiko Kishi (Hokkaido University, Sapporo, Japan), Nobuo Yaegashi (Tohoku University, Sendai, Japan), Koichi Hashimoto (Fukushima Medical University, Fukushima, Japan), Chisato Mori (Chiba University, Chiba, Japan), Shuichi Ito (Yokohama City University, Yokohama, Japan), Zentaro Yamagata (University of Yamanashi, Chuo, Japan), Hidekuni Inadera (University of Toyama, Toyama, Japan), Michihiro Kamijima (Nagoya City University, Nagoya, Japan), Takeo Nakayama (Kyoto University, Kyoto, Japan), Hiroyasu Iso (Osaka University, Suita, Japan), Masayuki Shima (Hyogo College of Medicine, Nishinomiya, Japan), Youichi Kurozawa (Tottori University, Yonago, Japan), Narufumi Suganuma (Kochi University, Nankoku, Japan), Koichi Kusuhara (University of Occupational and Environmental Health, Kitakyushu, Japan), and Takahiko Katoh (Kumamoto University, Kumamoto, Japan).

## Ethics approval

The study protocol was approved by the Ministry of the Environment's Institutional Review Board on Epidemiological Studies as well as the ethics committees of all participating institutions. All the participants provided written informed consent.

## Consent for publication

The authors have agreed with the publication.

## Declaration of competing interest

The authors declare that they have no competing interests related to the contents of this study.

## Appendix A Supplementary data

Fig. S1 Study flow chart. Fig. S1

Fig. S2 Allergic symptom combinations confirmed by caregiver-reported physician diagnosis. Fig. S2

Fig. S3 Venn diagram of allergic symptom combinations (atopic dermatitis, food allergy, asthma and allergic rhinitis) reported by caregivers as diagnosed by a physician. Fig. S3
