# Supplementary figures and images for: Allergy and immunology in young children of Japan: The JECS cohort
Source: World Allergy Organ J. 2020 Nov 7;13(11):100479. doi: 10.1016/j.waojou.2020.100479 (PMC7652713; doi:10.1016/j.waojou.2020.100479)

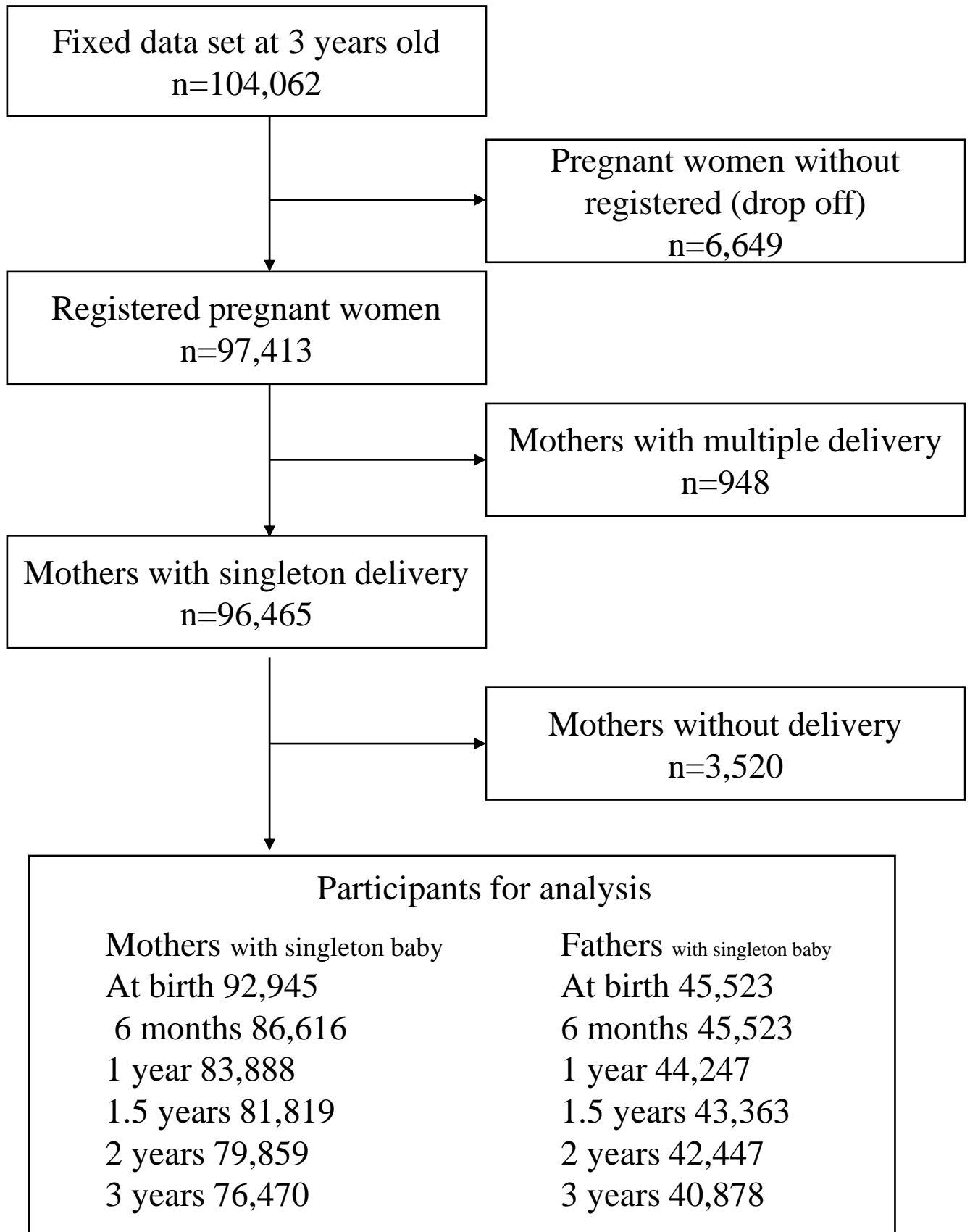

Supplement: Fig. S1 — Study flow chart. [file mmc1.pdf]

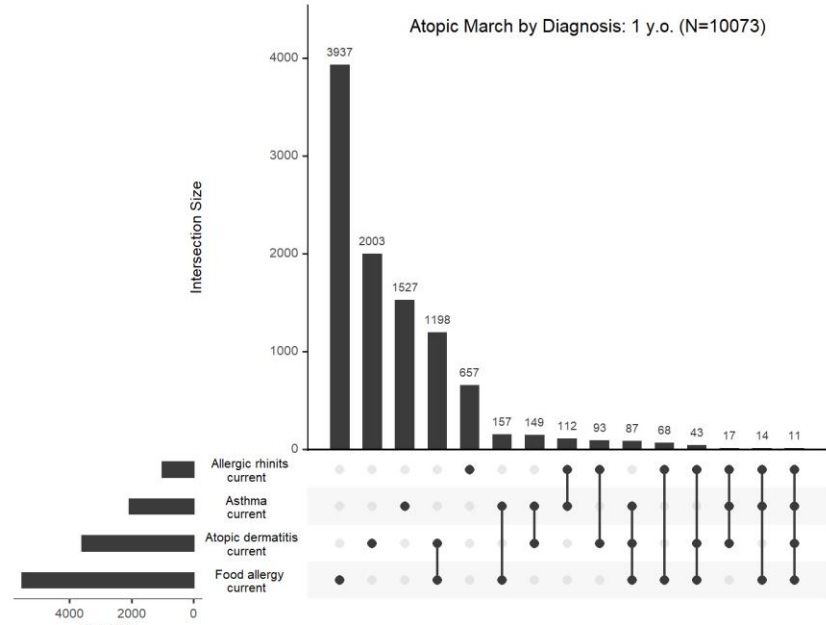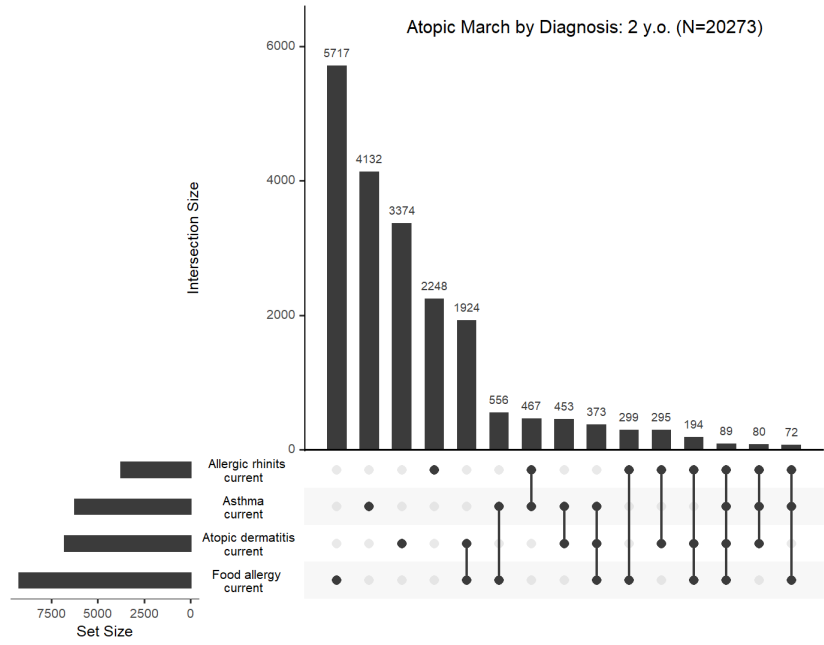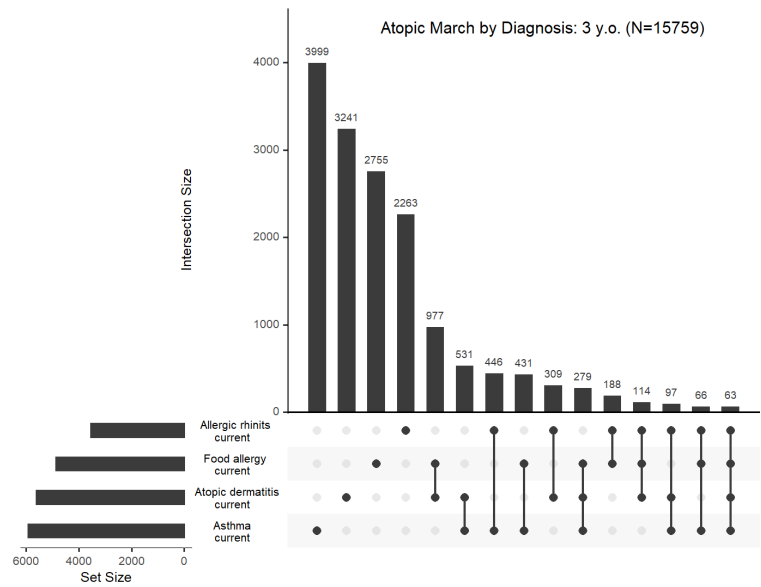

Supplement: Fig. S2 — Allergic symptom combinations confirmed by caregiver-reported physician diagnosis. [file mmc2.pdf]

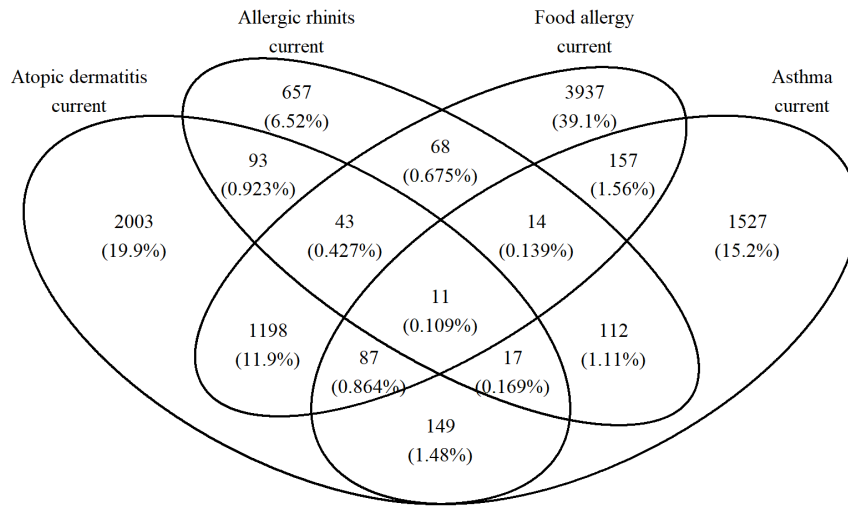

Atopic March by Diagnosis: 2 y.o. (N=20273)

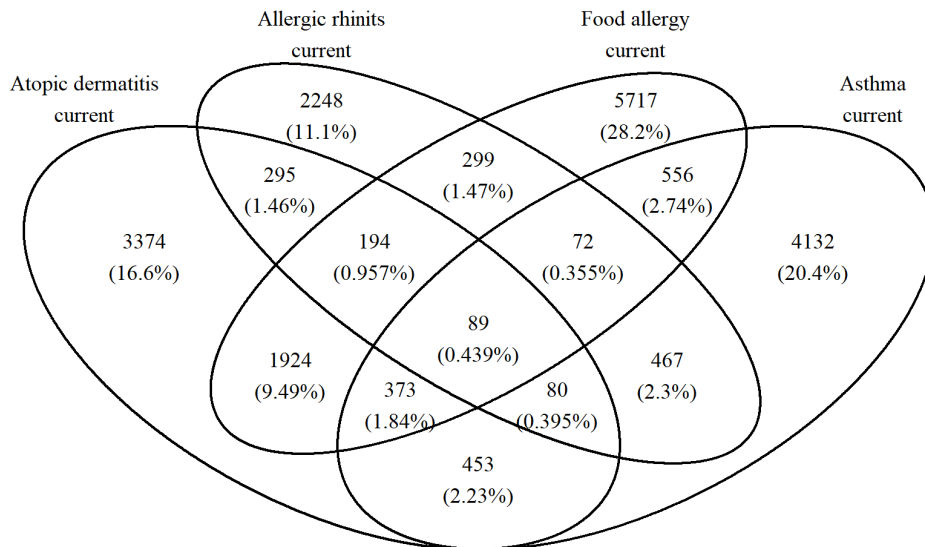

Atopic March by Diagnosis: 3 y.o. (N=15759)

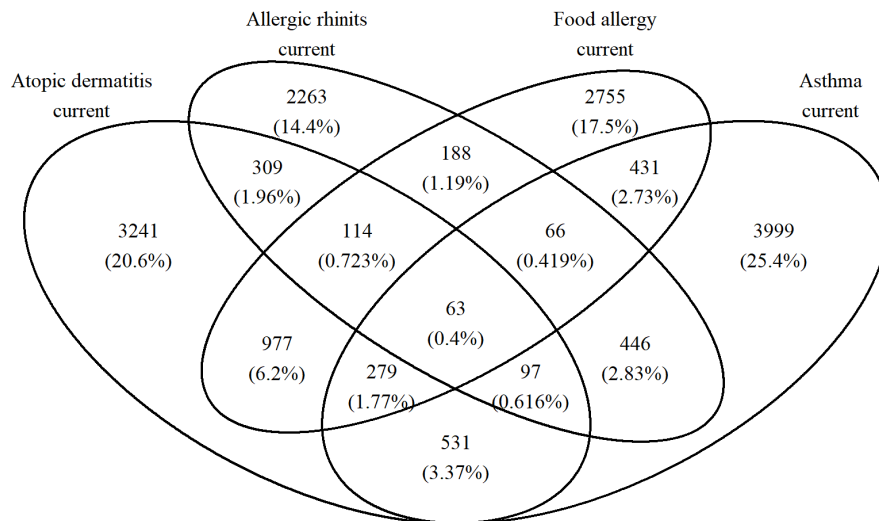

Supplement: Fig. S3 — Venn diagram of allergic symptom combinations (atopic dermatitis, food allergy, asthma and allergic rhinitis) reported by caregivers as diagnosed by a physician. [file mmc3.pdf]
