# Supplementary material for: Allergy and immunology in young children of Japan: The JECS cohort
Source: World Allergy Organ J. 2020 Nov 7;13(11):100479. doi: 10.1016/j.waojou.2020.100479 (PMC7652713; doi:10.1016/j.waojou.2020.100479)
Supplement: Multimedia component 4 [file mmc4.docx]

Table S1. Characteristics of study participants.

| Mothers |  |  | N=92945 |
| --- | --- | --- | --- |
|  | Study prefectures, n (%) | Hokkaido | 7463 (8.0) |
|  |  | Miyagi | 8233 (8.9) |
|  |  | Fukushima | 12157 (13.1) |
|  |  | Chiba | 5522 (5.9) |
|  |  | Kanagawa | 6039 (6.5) |
|  |  | Yamanashi | 6601 (7.1) |
|  |  | Toyama | 5021 (5.4) |
|  |  | Aichi | 5166 (5.6) |
|  |  | Kyoto | 3646 (3.9) |
|  |  | Osaka | 7184 (7.7) |
|  |  | Hyogo | 4720 (5.1) |
|  |  | Tottori | 2769 (3.0) |
|  |  | Kochi | 6366 (6.8) |
|  |  | Fukuoka | 6905 (7.4) |
|  |  | Kumamoto | 2703 (2.9) |
|  |  | Miyazaki | 1655 (1.8) |
|  |  | Okinawa | 795 (0.9) |
|  | Maternal age at recruitment, years | Mean ± standard deviation | 30.7 ± 5.1 |
|  |  | Minimum | 14 |
|  |  | 25% tile | 27 |
|  |  | 50% tile | 31 |
|  |  | 75% tile | 34 |
|  |  | Maximum | 48 |
|  | Past physician diagnosis, n (%) | Asthma | 10050 (11.0) |
|  |  | Hay fever, allergic rhinitis | 33089 (36.1) |
|  |  | Atopic dermatitis | 14509 (15.8) |
|  |  | Allergic conjunctivitis | 9159 (10.0) |
|  |  | Food allergy | 4454 (4.9) |
|  |  | Drug allergy | 2394 (2.6) |
|  |  | Urticaria | 14031 (15.3) |
|  |  | Contact dermatitis | 1771 (1.9) |
|  |  | Sick house syndrome | 305 (0.3) |
|  |  | Multiple chemical sensitivity syndrome | 128 (0.1) |
|  | Highest education completed, n (%) | Middle school | 4362 (4.7) |
|  |  | High school | 28470 (30.6) |
|  |  | Technical school | 1492 (1.6) |
|  |  | College of technology | 20715 (22.3) |
|  |  | Junior college | 15993 (17.2) |
|  |  | University | 18413 (19.8) |
|  |  | Graduate school | 1334 (1.4) |
|  |  | Unknown | 2166 (2.3) |
| Fathers |  |  | N=48081 |
|  | Study prefectures, n (%) | Hokkaido | 2659 (5.5) |
|  |  | Miyagi | 3849 (8.0) |
|  |  | Fukushima | 8181 (17.0) |
|  |  | Chiba | 3585 (7.5) |
|  |  | Kanagawa | 2320 (4.8) |
|  |  | Yamanashi | 4655 (9.7) |
|  |  | Toyama | 3054 (6.4) |
|  |  | Aichi | 2382 (5.0) |
|  |  | Kyoto | 2905 (6.0) |
|  |  | Osaka | 2790 (5.8) |
|  |  | Hyogo | 1762 (3.7) |
|  |  | Tottori | 1070 (2.2) |
|  |  | Tochi | 2210 (4.6) |
|  |  | Fukuoka | 3480 (7.2) |
|  |  | Kumamoto | 1653 (3.4) |
|  |  | Miyazaki | 1203 (2.5) |
|  |  | Okinawa | 323 (0.7) |
|  | Paternal age at recruitment, years | Mean ± standard deviation | 30.6 ± 5.0 |
|  |  | Minimum | 14 |
|  |  | 25% tile | 27 |
|  |  | 50% tile | 31 |
|  |  | 75% tile | 34 |
|  |  | Maximum | 47 |
|  | Physician diagnosis, n (%) | Asthma | 5124 (10.9) |
|  |  | Hay fever, allergic rhinitis | 14203 (30.3) |
|  |  | Atopic dermatitis | 5282 (11.3) |
|  |  | Allergic conjunctivitis | 2019 (4.3) |
|  |  | Food allergy | 1544 (3.3) |
|  |  | Drug allergy | 415 (0.9) |
|  |  | Urticaria | 4473 (9.5) |
|  |  | Contact dermatitis | 253 (0.5) |
|  |  | Sick house syndrome | 85 (0.2) |
|  |  | Multiple chemical sensitivity syndrome | 30 (0.1) |
|  | Highest graduation, n (%) | Middle school | 6512 (7.0) |
|  |  | High school | 33102 (35.6) |
|  |  | Technical school | 1948 (2.1) |
|  |  | College of technology | 16544 (17.8) |
|  |  | Junior college | 1855 (2.0) |
|  |  | University | 26136 (28.1) |
|  |  | Graduate school | 4088 (4.4) |
|  |  | Unknown | 2760 (3.0) |
| Children | Sex, n (%) | Boy | 47680 (51.3) |
|  |  | Girl | 45247 (48.7) |
|  |  | Unknown | 18 (0.0) |
|  | Annual household income, yen, n (%) | <2,000,000 | 4795 (5.2) |
|  |  | 2,000,000–3,999,999 | 29177 (31.4) |
|  |  | 4,000,000–5,999,999 | 27962 (30.1) |
|  |  | 6,000,000–7,999,999 | 13531 (14.6) |
|  |  | 8,000,000–9,999,999 | 5621 (6.0) |
|  |  | 10,000,000–11,999,999 | 2066 (2.2) |
|  |  | 12,000,000–14,999,999 | 817 (0.9) |
|  |  | 15,000,000–19,999,999 | 463 (0.5) |
|  |  | ≥20,000,000 | 279 (0.3) |
|  |  | Unknown | 8234 (8.9) |

Table S2. Nutritional status of children (N=92945).

|  |  | 6 months, n (%) | 1 year, n (%) | 2 years |
| --- | --- | --- | --- | --- |
| Breastfeeding without milk | Yes | 47188 (50.8) | 37113 (39.9) |  |
| Mixed feeding (breastfeeding and milk) | Yes | 19854 (21.4) | 12731 (13.7) |  |
| Milk feeding only | Yes | 15980 (17.2) | 26346 (28.3) |  |
| Timing of weaning | Minimum, 25^th^, 50^th^, and 75^th^ %ile, Maximum | - | 0, 5, 6, 6, 15 |  |
| Duration of breast feeding | Minimum, 25^th^, 50^th^, and 75^th^ %ile, Maximum |  |  | 0, 11, 14, 18, 38 |
| Rice | 3 months and earlier | 352 (0.4) | - |  |
|  | 4 months | 2781 (3.0) | - |  |
|  | 5 months | 50787 (54.6) | - |  |
|  | 6 months | 9232 (9.9) | - |  |
|  | 7–8 months | - | 15829 (17.0) |  |
|  | 9–10 months | - | 889 (1.0) |  |
|  | 11–12 months | - | 126 (0.1) |  |
|  | Not yet | 1540 (1.7%) | 14 (0.0) |  |
| Wheat | 3 months and earlier | 64 (0.1%) | - |  |
|  | 4 months | 476 (0.5%) | - |  |
|  | 5 months | 7636 (8.2%) | - |  |
|  | 6 months | 4720 (5.1%) | - |  |
|  | 7–8 months | - | 43156 (46.4) |  |
|  | 9–10 months | - | 13546 (14.6) |  |
|  | 11–12 months | - | 2017 (2.2) |  |
|  | Not yet | 41647 (44.8%) | 933 (1.0) |  |
| Soy | 3 months and earlier | 85 (0.1%) | - |  |
|  | 4 months | 570 (0.6%) | - |  |
|  | 5 months | 11247 (12.1%) | - |  |
|  | 6 months | 6108 (6.6%) | - |  |
|  | 7–8 months | - | 41771 (44.9) |  |
|  | 9–10 months | - | 10572 (11.4) |  |
|  | 11–12 months | - | 1558 (1.7) |  |
|  | Not yet | 37043 (39.9%) | 497 (0.5) |  |
| Fruits | 3 months and earlier | 1584 (1.7%) | - |  |
|  | 4 months | 4064 (4.4%) | - |  |
|  | 5 months | 20790 (22.4%) | - |  |
|  | 6 months | 6250 (6.7%) | - |  |
|  | 7–8 months | - | 33758 (36.3) |  |
|  | 9–10 months | - | 12140 (13.1) |  |
|  | 11–12 months | - | 2580 (2.8) |  |
|  | Not yet | 25667 (27.6) | 649 (0.7) |  |
| Milk | 3 months and earlier | 76 (0.1) | - |  |
|  | 4 months | 332 (0.4) | - |  |
|  | 5 months | 3634 (3.9) | - |  |
|  | 6 months | 2999 (3.2) | - |  |
|  | 7–8 months | - | 31038 (33.4) |  |
|  | 9–10 months | - | 26002 (28.0) |  |
|  | 11–12 months | - | 11268 (12.1) |  |
|  | Not yet | 47030 (50.6) | 6393 (6.9) |  |
| Hen egg | 3 months and earlier | 44 (0.0) | - |  |
|  | 4 months | 286 (0.3) | - |  |
|  | 5 months | 2774 (3.0) | - |  |
|  | 6 months | 2655 (2.9) | - |  |
|  | 7–8 months | - | 35194 (37.9) |  |
|  | 9–10 months | - | 26249 (28.2) |  |
|  | 11–12 months | - | 7301 (7.9) |  |
|  | Not yet | 48036 (51.7) | 5624 (6.1) |  |
| Fish | 3 months and earlier | 36 (0.0) | - |  |
|  | 4 months | 348 (0.4) | - |  |
|  | 5 months | 8537 (9.2) | - |  |
|  | 6 months | 5540 (6.0) | - |  |
|  | 7–8 months | - | 45141 (48.6) |  |
|  | 9–10 months | - | 16678 (17.9) |  |
|  | 11–12 months | - | 2846 (3.1) |  |
|  | Not yet | 40670 (43.8) | 745 (0.8) |  |
| Peanut | 6 months | - | 96 (0.1) |  |
|  | 7–8 months | - | 520 (0.6) |  |
|  | 9–10 months | - | 1332 (1.4) |  |
|  | 11–12 months | - | 1938 (2.1) |  |
|  | Not yet | - | 77530 (83.4) |  |

-: not evaluation

Table S3. Definitions of caregiver-reported physician diagnosis of outcomes.

| Outcome | Definition |
| --- | --- |
| Physician-diagnosed food allergy | A positive answer from the caregiver to the question: “Has your child ever been diagnosed by a physician as having food allergy in the past 12 months?” |
| Physician-diagnosed atopic dermatitis | A positive answer from the caregiver to the question: “Has your child ever been diagnosed by a physician as having atopic dermatitis in the past 12 months?” |
| Physician-diagnosed asthma | A positive answer from the caregiver to the question: “Has your child ever been diagnosed by a physician as having asthma in the past 12 months?” |
| Physician-diagnosed allergic rhinoconjunctivitis | A positive answer from the caregiver to the question: “Has your child ever been diagnosed by a physician as having allergic rhinoconjunctivitis in the past 12 months?” |
| Physician-diagnosed Kawasaki disease | A positive answer from the caregiver to the question: “Has your child ever been diagnosed by a physician as having Kawasaki disease in the past 12 months?” |
| Physician-diagnosed primary Immunodeficiency | A positive answer from the caregiver to the question: “Has your child ever been diagnosed by a physician as having primary immunodeficiency?” |

Table S4. Definitions of food allergy based on caregiver questionnaire responses.

| Outcome | Definition |
| --- | --- |
| Immediate food allergy | An affirmative answer from the caregiver to the question: “Has your child developed any allergic symptoms within 3 hours of eating certain foods and eliminated eating certain foods?” |
| Gastrointestinal allergy (non-IgE mediated food allergy) | An affirmative answer from the caregiver to the question: “Has your child ever had gastrointestinal allergy?” |
| Symptoms of gastrointestinal allergy (non-IgE mediated food allergy) | An affirmative answer from the caregiver to the question: “After eating certain foods, does your child have symptoms such as repeated vomiting, bloody stool, diarrhea, and weight loss starting after 3 hours to several days? This excludes food poisoning, gastroenteritis resulting from infection, and overeating.” |
